# Realistic details impact learners independently of split-attention effects

**DOI:** 10.1007/s10339-022-01123-z

**Published:** 2023-01-09

**Authors:** Alexander Skulmowski

**Affiliations:** Digital Education, Institute for Informatics and Digital Education, Karlsruhe University of Education, Bismarckstr. 10, 76133 Karlsruhe, Germany

**Keywords:** Realism, Learning, Visualization, Cognitive load, Attention

## Abstract

Realistic visualizations are considered to introduce the risk of distracting learners from relevant information. In two experiments, the interplay between realism and a known form of distraction, the split-attention effect, were investigated. This effect describes that spatially separating relevant information can have a substantial negative effect on learning. The experiments were conducted using short anatomy learning tasks to test whether a combination of realism and split attention would lead to the worst retention performance or, alternatively, whether realism can counteract the negative effects of split attention. The first experiment (*n* = 125) revealed that realism attenuated the cognitive load induced by split attention, suggesting a compensatory effect of realism (i.e., realism may have helped learners to deal with the detrimental influence of split attention). However, retention performance was not impacted in a similar way, indicating that this compensatory effect on subjective cognitive load may actually be the result of learners’ illusion that realistic details are helpful. Split attention significantly reduced retention performance. Experiment 2 (*n* = 152) resulted in negative effects of realism and split attention on retention. In sum, the experiments suggest that realistic details can affect learners independently of other visual design factors as exemplified by the split-attention effect. Thus, the assumption that realism is likely to distract learners is rendered implausible by the experiments, as the distraction of split attention should have amplified any distractive potential of realistic details. However, the results also suggest that the effects of realism on learning are still somewhat unpredictable.

## Introduction

Realistic visualizations are becoming increasingly important for digital learning. Realism is a central component of emerging technologies such as virtual reality, but can also often be found in online learning and other types of instructional materials. Due to this ubiquity, it is necessary to gain an understanding of how realism is processed during perception and learning. The two experiments in this paper investigate whether realistic details act in a negative way by distracting learners from other relevant information in learning tasks, such as text labels.

### Realism as a distracting element during learning

From a technical perspective, realism in computer-generated instructional visualizations has been described as a combination of the components of geometry, shading, and rendering (GSR; Skulmowski et al. 2022). In this definition, three-dimensional models in visualizations and real-time displays consist of a *geometry* with a variable level of detail that can be *shaded* by applying image textures. Finally, models can be *rendered* in more or less realistic styles. One of the reasons that speak against the use of realism (and that is often cited as the reason for negative results, e.g., Scheiter et al. 2009) is the potential for distraction inherent in detailed, and thus potentially demanding, visualizations. Realism has been alleged to feature a distracting quality for some time already. Dwyer (1976) summarizes different positions toward realism in visualizations and the warning that “excessive” realism may be counterproductive. Naturally, the question arises which level of realism is to be considered excessive and which may be declared as the optimum. Recent research suggests that these questions are task-dependent. For instance, a study on anatomy learning found that learning with realistic visualizations mainly has positive effects if the learning test makes use of the experience with the realistic representation, such as if the images used in a learning tests were also prepared in a realistic style (Skulmowski and Rey 2021). Moreover, two studies have revealed that higher levels of realism can result in better retention performance (or better performance for parts of a visualization that were rendered realistically), but at the same time increase subjective cognitive load (Skulmowski 2022a, b; Skulmowski and Rey 2020). However, the desire to explore objects has been found to increase with higher levels of perceived realism (Höst et al. 2022), providing another potential advantage of realistic visuals for learning. It should be considered that most positive effects of realism on learning pertain to the learning of shapes, while the learning of processes and abstract knowledge appears not to benefit from realistic details (Skulmowski et al. 2022).

Beyond visualizations, virtual reality is an important technology that heavily relies on a high level of realism to provide users with the illusion of being in a virtual world (e.g., Assländer et al. 2020; Kwon et al. 2013). It has been argued that in the long run, the specific functionalities of virtual reality (such as enabling experiential learning) will have a greater impact on learners than the mere realism of the virtual environment (Dalgarno and Lee 2010). This view is supported by a recent study on spatial learning in which object locations in a scene needed to be memorized in a virtual world (Huang and Klippel 2020). The study did not result in significant differences in task performance depending on visual realism, but revealed that a lower level of realism attracted more visual attention, leading the authors to assume an advantage of realism (Huang and Klippel 2020). Interestingly, a recent study found that instructional principles that have been well-established within other media do not necessarily need to be transferable to learning in virtual reality. Liberman and Dubovi (2022) established that simultaneously using multiple sensory modalities, in their case the visual and auditory senses to present text either as narration or on-screen, does not improve learning in virtual reality despite positive results of this approach for other types of media (Moreno and Mayer 1999). Thus, the remainder of this overview will be focused on learning with visualizations rather than with entire virtual environments.

Contrary to the positive effects of realism found in the literature, Smallman and St. John (2005) introduced the notion of “naive realism,” a term referring to the tendency of laypeople to prefer realistic visualizations over abstract representations in a range of tasks. According to Smallman and St. John, people generally assume a superiority of realistic displays as a result of a lack of knowledge of the perceptual system and due to being influenced by the marketing promises of computer technology. They claim that many people think that a more precise and realistic display will benefit performance, as laypeople mistakenly believe that this higher precision automatically results in a similarly detailed mental representation that they can draw upon. Thus, Smallman and St. John (2005) summarize that people may overestimate the usefulness of realistic imagery. After reviewing the conflicting results concerning performance data in the realism literature, we will now turn to gaze data in order to get a clearer picture of the processes underlying these results.

In an eye-tracking study, Lin et al. (2017) found that realistic visualization of the visual pathway (including the brain) drew more attention than a schematic visualization. This was indicated by a faster fixation of the realistic rather than the schematic versions and a longer dwell time on the former type of visualizations (Lin et al. 2017). Remarkably, the difference between the two visualizations was not as high as in many of the other studies discussed above. The realistic visualization of the brain did contain the gyri, thus giving this version a more complex appearance. However, all of the structures of the brain were presented in a solid color with subtle shading and contour lines; there were no discolorations, shiny highlights, or other visual components that could be expected to capture viewers’ attention. The schematic version only featured an outline of the brain without additional details. Both versions included the visual pathway as a simple line diagram. Thus, we have evidence to assume that even medium levels of realism (without “excessive” details) can grab viewers’ attention. Yet, no difference in learning performance based on the level of realism was found in that study.

Previous instructional eye tracking research has revealed that it can be advantageous for learners to revisit visualizations when learning with an accompanying text as a means to integrate verbal and visual content (e.g., Mason et al. 2013). Both Lin et al. (2017) and Mason et al. (2013) conclude that the schematic versions used in their studies promoted gaze patterns indicating a stronger integration of the texts and visualizations. In both cases, this effect was not detected for the realistic versions. From this result pattern, Lin et al. (2017) conclude that realistic visualizations may not be as effective in letting learners focus on relevant information. However, it is doubtful whether these results already constitute definite evidence for a negative, distracting effect of realism on learning. In order to answer this question, we will turn to a related effect from the field of multimedia learning, the *seductive detail effect*.

### Is realism a type of seductive detail?

The seductive detail effect was introduced by Harp and Mayer (1998) based on four studies in which participants learned using texts. In their first three experiments, the presentation of (visual) information that is interesting, but not relevant for the learning task lowered learning performance. Their fourth experiment showed that this negative effect is not as pronounced if the seductive details are placed at the end of a text passage to be learned, but can affect learners even more negatively if seductive details are located at the beginning of the passage. Harp and Mayer (1998) consider three explanations for their effect: (1) distraction: seductive details distract learners’ attention; (2) disruption: seductive details disrupt the connections within the learning material and hinder learners to establish a coherent schema; (3) diversion: seductive details activate unrelated mental models in learners’ minds that are then incorrectly used as the basis for their mental schema. Harp and Mayer (1998) interpret their results as a confirmation of the diversion hypothesis.

In the years that followed, the seductive details effect has been established as an important design effect within the field of multimedia learning (for meta-analyses, see Rey 2012; Sundararajan and Adesope 2020). However, there has been considerable debate over the cognitive mechanism behind the effect, leading to a controversy regarding the explanatory merits of the three aforementioned hypotheses (for an overview, see Bender et al. 2021). Meta-analyses and overviews generally state that the literature contains evidence for all three explanations of the seductive detail effect (e.g., Bender et al. 2021; Rey 2012). However, a recent study by Wang et al. (2021) found that the effect only impairs learning if the perceptual load of a task is already high (see Skulmowski et al. 2022, for a discussion on the aspect of perceptual load in the context of realism). It may suffice for our purposes to acknowledge the basic similarities between seductive details and realism that consist of a distractive effect, compelling learners to focus on potentially irrelevant information (see, e.g., Belenky and Schalk 2014; Lin et al. 2017).

As the distractive potential of realism is of key importance for guidelines on the design of visualizations, this paper presents two studies aimed at testing whether realism can indeed act as a distracting element. In order to achieve these goals, the studies utilize the *split-attention effect* to assess the influence of realism on the simultaneous processing of pictorial and textual information.

### Split-attention as a means to test the distraction hypothesis in simultaneous processing

The *split-attention effect* (e.g., Chandler and Sweller 1991, 1992) is one of the most important examples on how to avoid cognitive load through optimized design. It was built on the assumptions of cognitive load theory (Sweller et al. 1998, 2019), which posits that there are two major types of cognitive load competing for learners’ working memory capacity: *intrinsic cognitive load* (ICL), constituted by the number of elements to be learned (and their interactions), and *extraneous cognitive load* (ECL), resulting from irrelevant demands that learners need to cope with (Sweller et al. 2019). The split-attention effect is a prime example for how the reduction of ECL can benefit learners. This effect describes how learners must perform unnecessary visual searches when related content in learning material is not located spatially adjacent, but placed at separated locations (Kalyuga et al. 1999; Sweller 1989; see also the *spatial contiguity effect*, Mayer et al. 1995). A typical example of this effect would be to have a visualization that requires learners to look up the names of labeled parts in another location, such as below the visualization or on another page (see Chandler and Sweller 1992; Mayer et al. 1995). As searching for the various related elements at different locations does not foster, but rather interferes with learning, creating such a design has been identified as an induction of ECL (Ginns 2006).

Due to the visual nature of split attention, it appears to be a relevant effect to juxtapose with realism. Considering the literature discussed above, there could be two interactions between realism and split attention: on the one hand, if realism was found to act as a (visually) distractive influence, the two design aspects combined could result in an amplifying effect in which realism exacerbates the negative influence of split attention. On the other hand, there could be a compensatory effect, if the details of realistic visualizations help learners to re-identify elements in the visualization they previously looked at before searching the corresponding textual information at another location. Given that realistic visualizations have been found to result in longer dwell times than schematic ones (Lin et al. 2017) and as realistic details have been described as helping learners to distinguish between different elements in visualizations (Skulmowski and Rey 2021), realism may counteract the negative effects of split attention by providing concrete information that is more distinct and may thus be easier to find again after searching for a displaced label text. Previous research found that color cues can compensate for distracting and unnecessary detail in realistic visualizations (Skulmowski and Rey 2018), underlining the plausibility of a compensatory effect in this case.

### The present studies

In the two experiments described in the following, split attention is induced in schematic and realistic visualizations. As split attention is expected to lower learning performance compared to an integrated version due to a distractive influence, a more realistic visualization should amplify this negative effect if the same visual routes of cognitive processing are being affected by the two design factors. As a result, this interaction effect between split-attention (split vs. integrated) and realism (schematic vs. realistic) is tested (H_1a_). An additional contrasting hypothesis is assessed in which realism has a compensatory effect on split attention by helping learners re-identify the visual elements they looked at before searching for the label texts located at a distance. Thus, the result should be an interaction effect between the two factors in which learning performance is lowered by split attention, but with a weaker negative effect on those learners using the realistic rather than the schematic visualization (H_1b_). For the variable of ECL, these result patterns should be inverted.

## Experiment 1

### Method

#### Participants and design

Since a recent meta-analysis of the split-attention effect resulted in an effect of *g* = 0.63 in favor of integrated presentations (Schroeder and Cenkci 2018), an effect size of *η*_p_^2^ = 0.06 was decided upon (power = 0.80), resulting in a target sample size of 125 (calculated using G*Power, Version 3.1.9.2; Faul et al. 2009). Of the participants who completed this study, 107 were female and 18 were male. Participants needed to be between 18 and 30 years old German native speakers with no or little prior knowledge regarding the content of the study. They also needed to be using a PC or laptop instead of a device with a smaller screen, such as smartphones and tablets, to be able to participate. The two quality control items asking after distractions through noise and technical difficulties (from Skulmowski and Rey 2020) needed to be answered with a negative response in order for a dataset to be considered as complete.

The 2 × 2 between-subjects design with the factors realism (schematic vs. realistic) and split attention (split vs. integrated) used block randomization for the group assignment. This resulted in an assignment of 31 participants to the condition with the schematic rendering and split-attention, 32 to the condition with the schematic visualization and the integrated presentation, 32 learned with the realistic version presented in the split-attention format, and 30 used the realistic rendering with integrated labels.

#### Materials

**Fig. 1:**
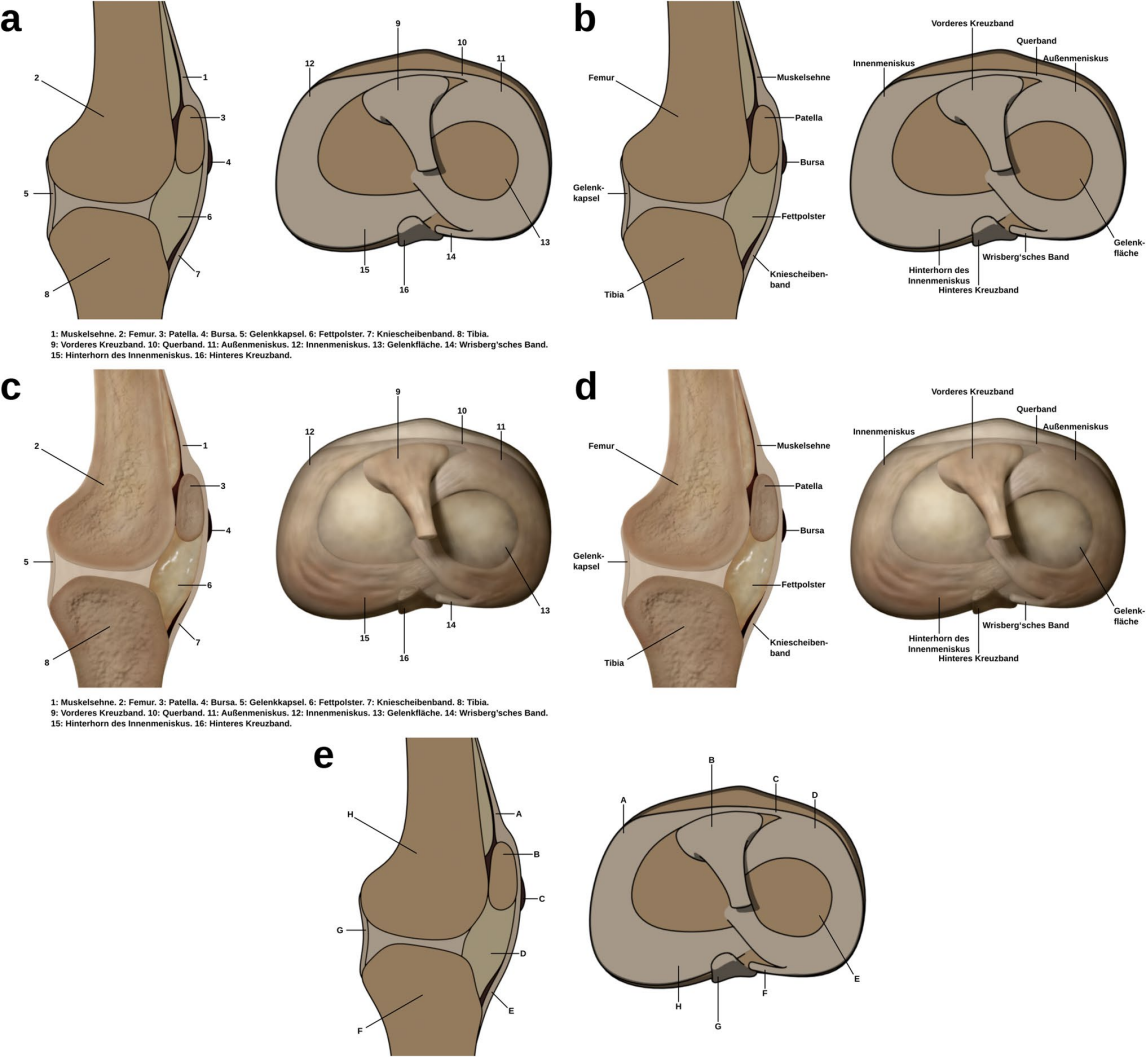
The instructional visualizations used in Experiment 1. The anatomy of the knee joint was to be learned. The visualizations are revised versions of the images used by Skulmowski and Rey (2020). **a**, **b** Displays the schematic versions of the visualization, while **c**, **d** presents the realistic version. **a**, **c** Shows the split-attention format, and **b**, **d** contains the integrated presentation. **e** features the two visualizations of the retention test. Adapted from Skulmowski and Rey (2020), Human Behavior and Emerging Technologies, 2, p 254 (https://doi.org/10.1002/hbe2.190). © 2020 Skulmowski and Rey (licensed under the Creative Commons Attribution License, http://creativecommons.org/licenses/by/4.0)

The study used learning materials from a previous study (Skulmowski and Rey 2020). The visualizations were revised using Blender (https://www.blender.org) by improving the lighting and contrast of the realistic version and changing the fill colors of the schematic version. The labeled instructional visualizations showed schematic (see Fig. 1a, b) or realistic (see Fig. 1c , d) renderings of the knee joint consisting of a side view (left) and a top view (right). Furthermore, the design factor of split attention was introduced by varying whether the label texts where placed at a distance from the components they belong to (see Fig. 1a, c) or located near the components in an integrated format (see Fig. 1b, d). The visualizations contained 16 items that were to be learned. The geometry of both realism levels did not differ, but the shading and rendering dimensions featured strong differences concerning their respective realism degree.

The learning tests (using the two visualizations shown in Fig. 1e, with a maximum score of 16) were performed utilizing sorting tasks in which name labels were dragged onto rectangles labeled with the corresponding letters seen in the test visualization. The schematic renderings were included for the tests. It was expected that the use of the realistic visualizations might induce biased results due to participants who were unfamiliar with the realistic version being potentially confused by some of the realistic details. Thus, the schematic version was chosen for the tests. The retention test had a reliability of McDonald’s *ω* = 0.86 (McDonald 1999). In addition, the three ECL items developed by Klepsch et al. (2017) were used, resulting in a reliability of McDonald’s *ω* = 0.93. The English translations of the original three ECL items are “During this task, it was exhausting to find the important information.”, “The design of this task was very inconvenient for learning.”, and “During this task, it was difficult to recognize and link the crucial information.” (Klepsch et al. 2017, p. 10), and these were modified as described by Skulmowski and Rey (2020) to ask participants concerning the visualizations rather than the entire task.

#### Procedure

The overall procedure was similar to the one used by Skulmowski and Rey (2020). Participants gave their informed consent, responded to the questions determining their eligibility (e.g., their native language), and received the instructions. In the instructions, they were informed that their task would be to memorize the names, shapes, and locations of the knee. Furthermore, they were informed about the time limit of 90 s during the following learning phase. Next, they were presented with the learning materials, cognitive load questions, a filler task, the learning tests (on one page), and an additional page of demographic questions and quality control questions. The filler task was included to prevent participants from relying on their phonological loop to answer the test as well as to minimize the danger of a ceiling effect. In the filler task, participants were asked to sort the 16 German federal states according to their number of day care centers with a time limit of 60 s. In this task, the 16 states were displayed on the right side of the screen and needed to be dragged onto a list on the left side in the correct order by the number of their day care centers. There were no further distractors in this task. The experiment was conducted online using SoSci Survey (Leiner 2021).

### Results

All analyses in this article were planned as 2 × 2 analyses of variance (ANOVAs). If one or more assumptions of this procedure were violated, nonparametric ANOVAs (Wobbrock et al. 2011) were computed instead.

#### Extraneous load

**Fig. 2:**
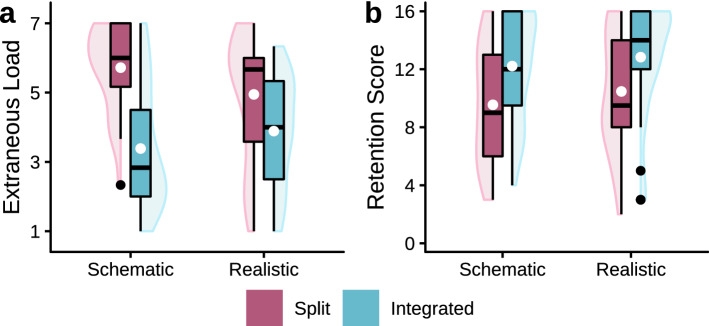
Boxplots with violin elements of the data from Experiment 1. **a** Displays the averaged ECL data and **b** presents the retention scores. White dots indicate the means of groups

An ANOVA of the ECL data (see Fig. 2a) confirmed H_1b_ with a significant interaction effect, *F*(1, 121) = 4.82, *p* = 0.030, *η*_p_^2^= 0.04. While a higher level of realism raised ECL, this already high ECL level was experienced as less strongly affected by the added demands of split attention. For those participants who learned using the schematic version, their “baseline” ECL was slightly lower in the integrated condition compared with the integrated realistic visualization. However, they were more strongly affected by the split attention demands than those who learned using the realistic version. This suggests that realism may have a compensatory effect. In addition, the ANOVA revealed a significant increase in ECL in the split attention presentation compared with the integrated format, *F*(1, 121) = 34.09, *p* < 0.001, *η*_p_^2 ^= 0.22.

#### Retention

A nonparametric ANOVA of the retention data only resulted in a strong negative effect of split attention, *F*(1, 121) = 14.28, *p* < 0.001, *η*_p_^2^= 0.11 (see Fig. 2b). The effect of realism did not reach significance despite a positive descriptive trend (*p* = 0.164) and the interaction effect also was not significant, *F*(1, 121) = 0.11, *p* = 0.740, *η*_p_^2 ^= 0.00.

## Experiment 2

The results of Experiment 1 did not produce conclusive evidence in favor or against H_1a_ and H_1b_. While the study revealed a significant compensatory effect of realism concerning ECL, this effect could not be observed in the retention data. Thus, a second experiment was conducted to assess the validity of the two hypotheses. Furthermore, some changes in the design logic behind the learning visualizations needed to be made.

### Method

#### Participants and design

In order to arrive at a more definite set of results, a number of improvements were made in the design of a new visualization. In addition to these changes, a more conservative effect size of *η*_p_^2^= 0.05 (power = 0.80) was used and led to a target sample size of 152. The participation and completion criteria were adapted from Experiment 1. Of the 152 participants whose datasets count as completed, 132 were female and 20 were male. The experiment had the same experimental design as Experiment 1. Block randomization assigned 40 participants to each of the two groups learning with an integrated format, 37 participants to the group using the schematic visualization in the split-attention format, and 35 used the realistic version in the split-attention format.

#### Materials

The learning materials used for this study followed the basic logic of the ones used in Experiment 1, but there were a number of differences. In the learning phase of Experiment 1, participants saw a side view and a top view of the knee. This aspect may have introduced some split attention already, therefore potentially weakening or otherwise biasing the results. In addition, the two views of the knee slightly differed regarding their dimensionality. While the side view presented essentially a flat cross section, the top view contained some dimensionality brought about by the shapes of several ligaments. These two aspects were made consistent in the materials used for Experiment 2.

**Fig. 3:**
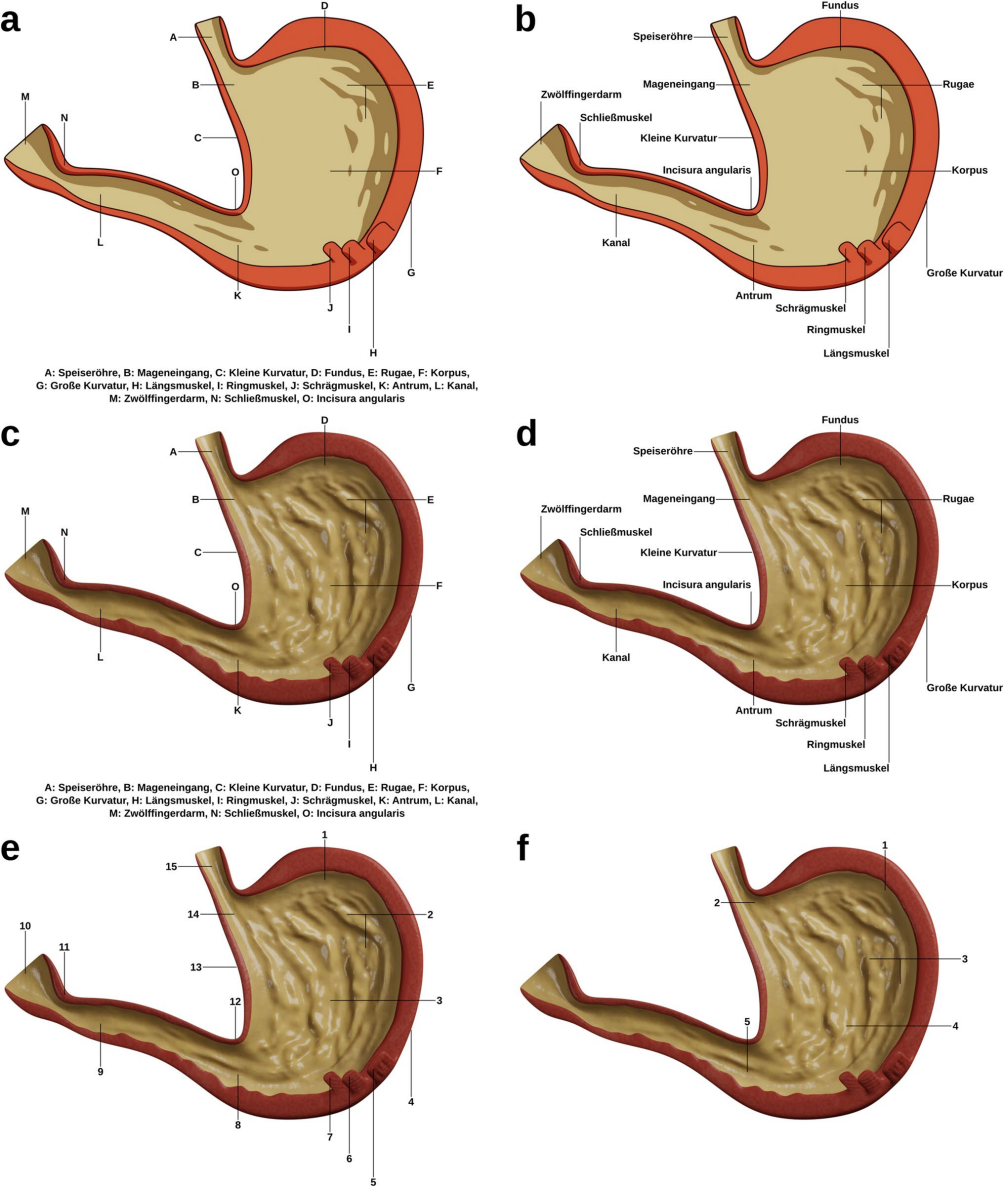
Instructional visualizations of the stomach were created for Experiment 2 using Blender 3.0.0 based on Gray (1918, pp. 1164–1165), with additional information from Betts (2013), Cole (2015), and other resources. **a**, **b** Shows the schematic version, and **c,** **d** display the realistic rendering. The format was contrasted using split-attention visualizations (**a,** **c**) and an integrated format (**b,** **d**). **e** Shows the retention test and **f** displays the near transfer test

The labeled instructional visualizations used for the experiment show an interior view of the stomach. They are schematic (see Fig. 3a, b) or realistic (see Fig. 3c, d) visualizations. The schematic version slightly differs concerning the geometry, as the realistic rendering contained some more irregular shapes to add detail. Through the glossy shading of the inside layer of the stomach, the realistic version featured much more detail. In addition, the realistic version showed some other details such as shaded muscle fibers. The shading and rendering dimensions contributed toward the more striking differences between the two visualizations. Like in Experiment 1, split attention was induced by placing the labels at a distance (see Fig. 3a, c), contrasted with an integrated format (see Fig. 3b, d). There were 15 labels and corresponding components to be learned. The study contains two learning tests, one retention test with a maximum score of 15 in which the connecting lines were placed exactly at the same locations as in the learning phase (see Fig. 3e, McDonald’s *ω* = 0.87) and a near transfer test with a maximum score of 5 in which the labels and connecting lines of those components exhibiting the highest ambiguity (due to unclear boundaries) were moved to other locations on the image (see Fig. 3f, McDonald’s *ω* = 0.74). The near transfer test was introduced in order to check whether participants were able to apply their knowledge if the order and placement of the labels is changed rather than relying on memorizing the order of the label texts in a (counter-)clockwise manner. Both tests were presented on the same page and used a series of drop-down menus to enable to select the corresponding label names from. The experiment used the same ECL items as Experiment 1 (McDonald’s *ω* = 0.94).

#### Procedure

The procedure was identical to Experiment 1, except that the learning phase lasted 60 s due to a lower number of labels with less difficult label texts. Also, the filler task had a different sorting task.

### Results

#### Extraneous load

**Fig. 4:**
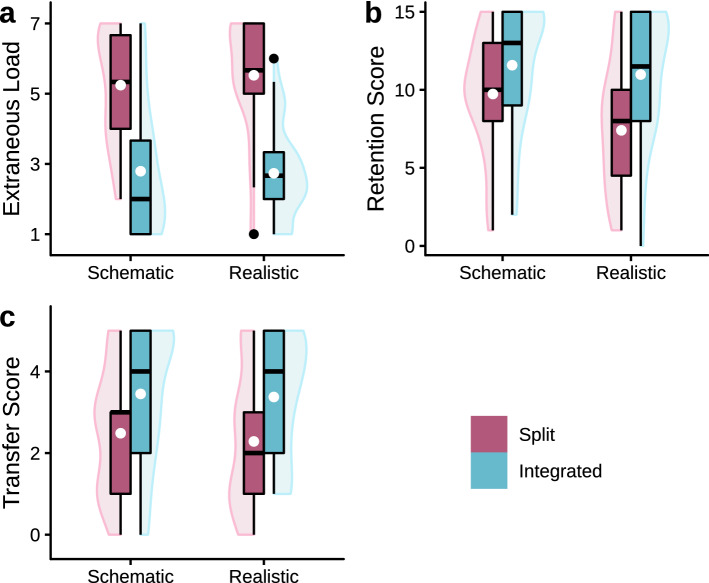
Boxplots with violin elements of the data from Experiment 2. **a** contains the averaged ECL ratings, **b** shows the retention scores, and **c** features the near transfer test data. White dots indicate the means of groups

A nonparametric analysis of the ECL data (see Fig. 4a) resulted in a significant negative effect of split attention, *F*(1, 148) = 103.91, *p* < 0.001, *η*_p_^2^= 0.41. However, the compensatory effect of realism on split attention could not be confirmed, *F*(1, 148) = 0.43, *p* = 0.511, *η*_p_^2 ^= 0.00, and there was no significant effect of realism (*p* = 0.397).

#### Retention

The retention test data (see Fig. 4b) were analyzed using a nonparametric ANOVA and revealed negative effects of split attention and realism, *F*(1, 148) = 20.92, *p* < 0.001, *η*_p_^2 ^= 0.12, and *F*(1, 148) = 6.93, *p* = 0.009, *η*_p_^2 ^= 0.05, respectively. There was no significant interaction effect, *F*(1, 148) = 2.45, *p* = 0.120, *η*_p_^2 ^= 0.02. However, it is noticeable that the realistic version presented in the split attention format elicited the lowest retention scores. Therefore, a compensatory effect on retention performance (H_1b_) seems unlikely to exist.

#### Near transfer

Regarding the near transfer data (see Fig. 4c), only the negative effect of split attention reached significance, *F*(1, 148) = 15.84, *p* < 0.001, *η*_p_^2 ^= 0.10, while neither the interaction effect, *F*(1, 148) = 0.03, *p* = 0.873, *η*_p_^2 ^= 0.00, nor the main effect of realism (*p* = 0.397) achieved significance.

## Discussion

The main research question underlying the experiments was whether realistic details are “seductive” and distract learners from relevant information. In two experiments, the interplay of realism and split attention were investigated to assess whether realistic details worsen the impact of suboptimal visual design or whether realism may be helpful to overcome obstacles of visual design during learning. In order to test the hypothesis that realism is visually distracting, another visual distractor was introduced: the split-attention effect. Based on the results of the two studies, there is no conclusive evidence that realism generally acts as a distracting element. In both experiments, the split-attention effect induced higher extraneous load ratings and lower retention performance for visualizations in which the labels were presented separated from the visuals. These results confirm that the spatial separation of related information can have a distracting influence on attentional and perceptual processes. Regarding realism, the result pattern is less clear. In Experiment 1, a higher level of realism did not significantly affect retention performance, though there was a tendency toward a positive effect on the descriptive level. Importantly, the study revealed an interesting interaction effect in which the realistic version presented in an integrated format already elicited higher extraneous load ratings than the schematic and integrated variant. However, the addition of the split presentation of labels resulted in a strong increase in extraneous load for the schematic version, but only in a slight increase for the realistic visualization. This could be regarded as supporting the idea of a compensatory effect of realism (H_1b_) and is also in line with warnings that realism may induce an overconfidence in the quality of visualizations (see Smallman and St. John, 2005). While learners may be able to recognize how split attention negatively affects them during learning, their judgment may become less accurate if realism is introduced, resulting in a more forgiving attitude toward the split design. However, Experiment 2 neither supports an amplifying (H_1a_) nor a compensatory (H_1b_) effect of realism. In contrast to the previous experiments (e.g., Skulmowski 2022a; Skulmowski and Rey 2021), a higher level of realism did not help, but diminished retention performance. The retention results of Experiments 1 and 2 do not support the claim that realism is able to worsen other visual demands that may exist in a visualization. Rather, it appears that realism acts independently of other (visual) design factors.

Despite evidence from several studies that learning shapes is generally enhanced by using realistic visualizations (Skulmowski et al. 2022), the two studies either showed null or negative effects of realism on learning. This could be a consequence of the learning materials not optimally using the potentials of realistic details. For instance, the realistic visualization used for Experiment 2 was filled with certain details that might provoke attention, such as glossy surfaces and finer surface detail. It might be the case that these details could indeed have had a negative influence on participants’ ability to quickly distinguish the relevant from the irrelevant information. Thus, the realistic details in Experiment 2 might have primarily acted as a cognitive cost without benefits of a comparable magnitude (see Skulmowski and Xu 2022, for an overview of cognitive cost–benefit models). This cost–benefit approach should be investigated further, but will probably require a more exact quantification of all aspects involved (including eye tracking data). Additional research regarding the specific visual qualities of different realistic visualizations and their influence on perception, attention, and learning is needed.

The experiments provide an investigation into the cognitive effects of realism and have a variety of implications for future research. Generally speaking, the studies revealed that the various design factors of visualizations appear to exert their influence independently without affecting each other.

### Limitations and outlook

Some limitations relevant to the interpretations of the results need to be considered. In both studies, the vast majority of participants was female, resulting from the gender distribution in the enrollment of the courses. Future research should test if there are gender-based differences in preferences and performance when learning with visualizations of different levels of realism. Furthermore, the learning tasks are rather short. As discussed in similar research (e.g., Skulmowski and Rey 2021), these short learning tasks can extend our knowledge regarding how visualizations need to be designed to be of use in lectures or other situations in which visualizations are presented only for a short period of time. In addition, the perceptual effects investigated in the two studies may have the most impact on learning if only a short period of time is given for the learning tasks, while learners might not be as strongly affected if they have more time to cope with the suboptimal design of visualizations. An older study on the issue of prior knowledge and realism found that learners with low or intermediate prior knowledge require more time to learn with realistic visualizations than those with high prior knowledge (Dwyer 1975). Thus, the effects of different learning times should be investigated in future studies.

The levels of realism compared in the studies are generally relatively similar. The schematic versions always made use of contour lines and solid colors for the different elements, while the realistic versions added some surface detail and utilized a high fidelity rendering method. Thus, the results of the studies are comparable and enable a systematic investigation. However, there needs to be more research conducted on the potential effects of other realism levels, such as visualizations created from high resolution scan data that provide much more detailed geometry and textures. Future studies should include such highly realistic visualizations.

Furthermore, it needs to be noted that the two studies used test materials of varying realism levels. While Experiment 1 features schematic test visualizations, Experiment 2 contains realistic rendering in the testing stage. The choice of non-biased test visualizations has been discussed in the literature (e.g., Skulmowski 2022a; Skulmowski and Rey 2018). Based on a previous study, learning with realistic visualizations can lead to particularly high retention scores if the test visualizations are equally realistic, while the use of schematic visualizations in the testing stage does not appear to favor those who learning using schematic representations (Skulmowski and Rey 2021). Thus, Experiment 1 may have resulted in an overall positive effect of realism if realistic renderings had been used in the tests. However, following the aforementioned results, Experiment 2 should have shown a positive overall effect of realism, as the tests used the realistic version of the visualization. In sum, it should be acknowledged that the two study designs did not feature a bias against learning with realistic visualizations, yet found a negative effect of realism in one experiment.

In addition, it should be noted that the studies in this paper have a strong focus on cognitive variables. However, the aspect of interest has been identified as an important variable both in research on seductive details (Wang and Adesope 2016) as well as realism in general (Belenky and Schalk 2014). Pictures can have an important effect on motivation (Lindner et al. 2017) that should also be investigated further in the context of realism.

Another limitation of the two studies to consider is that certain learner characteristics may have had an impact on the results. An important learner characteristic to consider when learning with 3D models is spatial knowledge. Spatial knowledge has been shown to affect how well learners can deal with the cognitive demands of (rotatable) 3D models (e.g., Huk 2006). However, in presentations of renderings that do not feature rotatable 3D models, spatial ability has not been found to be a significant influence on learning (Huk et al. 2010). As the 3D models used in the visualizations of the two present experiments were not rotatable by the participants (see Skulmowski 2022b) and are rather flat in their design (as most of the visualizations show cross sections), it is unlikely that spatial ability could have had a major impact in the studies. Furthermore, the sample sizes of the studies are large enough to assume that the spatial ability of the participants was sufficiently balanced in order to avoid a confounding influence of spatial ability. However, future studies on split attention in the context of realism should consider to include this aspect.

Similarly to spatial ability, the learner characteristic of prior knowledge has been found to affect learning with visualizations (Huk et al. 2010). In the two present studies, prior knowledge was assessed by a single question item asking the participants to indicate whether they had little or no prior knowledge concerning the respective body parts to be learned. Participants who did not confirm to have a low level of content domain knowledge were not included in the samples. This aspect could be assessed in more detail in future studies. However, given that the samples consisted of pre-service teachers, it is unlikely that the samples contained students with such a high level of prior knowledge that may have biased the studies. In addition, the sample sizes are sufficiently large that a balanced distribution of prior knowledge is probable.

As discussed above, results from studies on learning with visualizations do not necessarily provide evidence that an effect needs to be reproducible when learning in virtual reality (Liberman and Dubovi 2022). Thus, we need to be careful when attempting to transfer the results of the present studies to learning in virtual worlds. It may be the case that realistic virtual scenes are faster to process visually (Huang and Klippel 2020), therefore potentially lending themselves to more complex spatial arrangements that might induce split attention. In this case, realism may have a similar compensatory effect as in Experiment 1. Empirical studies are needed to confirm this hypothesis.

### Conclusion

In two experiments, the interplay between realism and split attention was investigated. Since the effects of these both factors were found to be independent of each other, it is unlikely that realism acts as a comparably strong force of visual distraction as split attention. One result suggests that learners may gain the impression that a suboptimally designed visualization featuring split attention may be perceived to be less demanding if the visualization is presented in a realistic style. This potential for inaccurate judgments due to the presence of realism should be considered in future research.

## Data Availability

The data of these experiments is available from the author upon request.
